# Metabolo-politics: Bovine Metabolism and Environmental Biopower

**DOI:** 10.1177/02632764261438395

**Published:** 2026-05-15

**Authors:** Jonathon Turnbull, George Cusworth, Adam Searle, Else Vogel, François Thoreau, Catherine Oliver

**Affiliations:** Durham University; University of Oslo; University of Nottingham; University of Amsterdam; University of Liège; Lancaster University

**Keywords:** metabolism, metabolo-politics, biopower, cows, carbon, nitrogen, agriculture

## Abstract

More-than-human metabolic processes increasingly constitute the locus of contemporary biopower, often responding to industrially-altered biogeochemical cycles. We conceptualise ‘metabolo-politics’ to examine the targeting of bodies and populations via interventions in the biochemical surroundings and ecologies that shape them; and simultaneously, the administration and modulation of materials in the environment via interventions in the bodies and populations that ingest, digest, and excrete them. Focusing on the governance of environmental crises related to carbon and nitrogen pollution, particularly in livestock sectors in the Global North, we offer three analytics for theorising metabolo-politics as a form of governance oscillating between bio-politics and anatomo-politics. They are: the *dispositive*, which grapples with the relations of matter and discourse that shape emergent problematisations of metabolism; the *milieu*, which is the more-than-human scene of intervention; and *technologies*, which are the diverse means through which power is operationalised metabolically and through which bodies, populations, and environments are governed.

## Introduction

Michel Foucault (2007: 1) defined biopower as the ‘set of mechanisms through which the basic biological features of the human species became the object of a political strategy’. Biopower has since provided a fruitful theoretical frame for analysing how power operates through diffuse networks of discourse and matter. Characterising a liberalising shift from sovereign power (making die and letting live) to biopower (making live and letting die), Foucault (1979: 139) detailed ‘two poles’ of biopower ‘around which the organisation of power over life was deployed’ in the 17th century. These ‘poles’ are an ‘*anatomo-politics* of the human body’, which focuses on the disciplining and optimisation of individual subjects; and a ‘*bio-politics of the population*’, concerned with the ‘species body’ and its larger-scale biological processes. Whilst scholarship tends to focus on either bio- or anatomo-politics as distinct strategies of liberal governance (e.g. Brown and Knopp, 2010), Foucault (1979: 139) stressed ‘these forms were not antithetical’. Instead, they ‘constituted rather two poles of development linked together by a whole intermediary cluster of relations’ (Foucault, 1979: 139).

This article examines a specific cluster of relations that mediate the scales, sites, and practices of environmental governance between anatomo-politics and bio-politics: those pertaining to *metabolism* and its increasing role in the governmental arrangement, transformation, and circulation of life. *Metabolo-politics*, we contend, oscillates between the poles of anatomo- and biopolitics, targeting the material circulations that bodies and populations both inhabit and help form. We conceptualise metabolo-politics as an emerging environmental mode of biopower where the regulation of material flows, along with the discursive and calculative practices that make those flows tractable, are mobilised to administer, govern, and manage life in circulation. Metabolo-politics does not replace other modes of biopower, though. Following Foucault’s (2007: 8) understanding of how modes of power emerge over time, ‘there is not a series of successive elements [in which] the appearance of the new [causes] the earlier ones to disappear’. Metabolo-politics thus operates simultaneously with other forms of biopower.

We draw on diverse qualitative, ethnographic, archival, and discursive empirics relating to post-industrial agriculture in the UK and the Netherlands that we, collaboratively or individually, have engaged in over the past five years of study (Cusworth et al., 2022b, 2024; Oliver, 2024; Oliver and Turnbull, 2021; Searle et al., 2024; Turnbull and Oliver, 2021; Vogel, 2025). In diverse ways, cattle are being subjected to novel regimes of biopower that aim to optimise their metabolic functioning as a means of managing the environments – such as local waterways and soil ecosystems, or the global atmospheric sink – that they help to create. This previous work examines the heated political, scientific, technological, and economic struggles over the future of agriculture, itself shaped by understandings of, and interventions in, the digestive capacities or consequences of livestock (see Beldo, 2017). Our theorisation of metabolo-politics is thus rooted in two similar technoscientific, post-industrial, and capitalist agricultural contexts. Yet we note its relevance beyond these contexts and, in conclusion, call for situated, provincialised (Chakrabarty, 2009) accounts of metabolic governance (Barua, 2025a; Davies, 2019, 2025; Meloni, 2020).

Recent scholarship has emphasised the underappreciated materialist and nonhuman concerns within Foucault’s writing (Gabrys, 2014; Lemke, 2015; Nealton, 2016; Philo, 2012; Usher, 2014). Whilst these interpretations have guided examinations of biopower enacted through ecological dynamics and more-than-human assemblages (e.g. Braun, 2007; Holloway et al., 2009; Srinivasan, 2013), they tend to describe dyadic relations between humans and nonhuman living beings (i.e. animals, microbes) as biopower’s object of governance. The specific focus of metabolo-politics, by contrast, is on the movement and transformation of materials as they are ingested, digested, and excreted (Barua, 2025a; 2025b; Landecker, 2013a; Mol, 2021; Solomon, 2016; Wilson, 2015). It concerns governance that exploits the permeability and metabolic co-constitution of bodies and environments.

Since the 19th century, metabolism has inspired scientists and philosophers to think beyond the ‘two-ness’ of organism and environment towards the ‘third thing’, and has thus been ‘a concept with which to move across and beyond – or simply hold in permanent oscillation – polarities of all kinds’ (Landecker, 2013a: 223). In the wake of contemporary environmental crises in the ‘aftermath’ of industrialisation, metabolism is increasingly the concern of public and private governance. This has prompted theoretical work to rethink power, materiality, and life, most notably through notions such as anthropogenic biology and biofallibility (Landecker, 2025; see Barua, 2025a; Hinchliffe and Ward, 2014; Hinchliffe et al., 2013). Our conceptualisation of metabolo-politics continues in this vein, drawing attention to emerging forms of governance that seek to address biochemical processes and ecologies that are implicated by human and nonhuman eating (Mol, 2021).

Herein, we describe a more-than-human mode of biopower enacted through the metabolic governance of life. We propose a conceptualisation of metabolo-politics that concerns the administration of bodies and populations via intervention in the flows that constitute their environmental milieu; and simultaneously, the administration, modulation, and canalisation of materials via intervention in the bodies and populations they flow through. This mode of biopower emphasises the transformative and porous way that bodies are metabolically immersed in their surroundings (Dupré and O’Malley, 2009) whilst maintaining a sensitivity to how distinctions between inside and outside, and body and environment, continue to be relevant for governance purposes. Bodies and populations are, of course, not newly being understood as porous, relational, and leaky (Hokowhitu, 2020; Lamoreaux, 2016; Meloni, 2021; Todd, 2016). Rather than enthusiastically proclaiming a new ontology of permeability and porosity, our aim is to rethink the relationship between power and life as metabolic porosity becomes a locus of governance.

Our interest lies in the governmental and scientific logics being deployed to capitalise on metabolism’s relational materiality for specific purposes. Our case studies aid us in theorising metabolo-politics across different scales (regional and global) within the Western European context, adding a *scalar* dimension to Barua’s (2025a) *spatial* approach. Our contribution is to sharpen the theoretical tools which lure attention to how specific metabolic processes are known and thus made governable, and to the uneven outcomes of this emerging manifestation of biopower.

## Empirical Groundings

Our article draws on two case studies related to the governance of biogeochemical cycles and biochemical processes. Biogeochemical cycles refer to the movement and transformation of chemicals and abiotic compounds like water, nitrogen, or carbon between living things, the Earth’s surface, and the atmosphere (Huang et al., 2024). Biochemical processes, on the other hand, refer to the chemical processes that occur within, or relate to, the corporeality of organisms. While these processes obviously overlap, the former gestures towards the planetary nature of the cycles in question, while the latter gestures towards the corporeal.

Our first case concerns the development of feed additives designed to reduce methano-genesis: the metabolic process in bovine rumina that produces the potent greenhouse gas methane, CH_4_. Such feed additives promise to lower livestock agriculture’s carbon emissions whilst improving feed conversion efficiency for environmental and economic gain. These developments are spearheaded by scientific and industry experts in the US, the UK, and Switzerland. They are mostly private initiatives and public-private research projects initiated in the 2000s and 2010s. The second regards the development of EU and national policies designed to reduce the contributions livestock systems make to nitrogen pollution in the Netherlands. We are particularly interested here in the regulation of cattle populations, farm size, and feed and waste management practices, along with the 2019 sectoral protests they catalysed. These two cases – which feature different circuits of capital, research, governance, and culture – highlight both the common features of metabolo-politics and its heterogeneity.

Given both these cases are located in the Global North (insofar as any aspect of the global food system *can* be located in one place), the specific instantiations of metabolo-politics they allow us to trace must be understood accordingly. Where previous scholarship has explored how (bovine) bodies and their metabolic lives are understood and engaged with differently in, for example, India (e.g. Barua, 2025a; Narayanan, 2023; Turnbull and Barua, 2023), Colombia (de la Cadena and Martínez Medina, 2020), and the UK (Cusworth et al., 2024), we hope to show here how differences exist even within geographies typically assumed to be economically, culturally, and governmentally similar. These methodological choices invite further comparison between different versions of metabolo-politics, rather than implying that European or Global North metabolic logics are themselves singular or that they constitute the sole manner in which metabolism is enrolled into the operations of biopower today.

Specific metabolic pathways within bovine bodies are implicated in highly contrasting modes of environmental governance, shaping ecologies at different spatial and temporal scales. Our examples show how interventions into bovine metabolisms affect both the transformation (e.g. efforts to reduce methane emissions through feed alteration) and circulation (e.g. waste management alterations) of matter (Barua, 2025a). The aim of this article, then, is to explore two specific metabolic processes that have become targets for governance in our field sites, which endeavour to cultivate economic value through life while remediating wider environments. Spurred by an analytical openness to what materials are and what they become, and to how power works, our attempt to read across two Global North technoscientific capitalistic modes of metabolo-politics highlights how ‘[d]ifferent knowledge practices enact various versions of metabolism’ which are ultimately ‘animated by different concerns and [imply] contrasting relations and courses of action’ (Vogel, 2025: 57).

Where our empirical engagements form the substance of our efforts to theorise a metabolo-political mode of biopower, Thomas Lemke’s writing on Foucault provides our conceptual structure. In *The Government of Things* (2021), Lemke explores the nascency of new materialist thought in Foucault’s work (see also Usher, 2014), identifying three analytics relevant to more-than-human or environmental analysis: dispositive, milieu, and technologies. Herein, we develop these concepts in a metabolic mode: (1) the metabolic dispositive grapples with the relations of matter and discourse that shape emergent problematisations of metabolism; (2) the metabolic milieu is the distributed more-than-human material context within which bodies and populations exist and the scene of intervention in which metabolo-political interventions are staged; and (3) metabolic technologies are the diverse means through which power is operationalised and bodies, populations, and flows are governed. These concepts are not discrete, nor does any one precede or wholly determine the others. They overlap and condition one another in iterative ways.

## Metabolic Dispositives

Different food system actors – from alternative farm movements, to big agri-business, to international political bodies – now frame environmental problems in metabolic terms (Searle et al., 2024). For these actors, metabolism offers both a lens through which socio-ecological issues are understood and a means through which they might be managed. This speaks to the emergence of a general metabolic dispositive: that metabolic dynamics have contributed to the manner in which diffuse material flows and biogeochemical cycles have gone awry, and that alterations to those metabolisms might help remediate them. Here, we see the emergence of metabolo-politics as part of a heightened politics of cycles (Hopwood et al., 2021), where the faltering progress of modernity is ascribed to rifts in a stable socio-ecological order (Clark and York, 2005). This general disposition finds articulation in relation to *specific issues*. We focus on carbon and nitrogen. These two distinct dispositives share a concern with the flow, transformation, and cycling of materials as they move ‘transversally’ (Barua, 2025a) through bovine (and other-than-bovine) bodies in intensive livestock systems. But they differ in terms of the scales they perform, the aspect of bovine metabolism they problematise, the publics they engage, and the type of interventions they inaugurate.

Foucault (1980: 194–5) defined the dispositive as a ‘heterogeneous ensemble’ that encompasses material and discursive elements: a ‘genesis’ that forms ‘at a given historical moment’ to respond to ‘an urgent need’. The dispositive names ‘the network of discourses, practices, and institutions, variable across space and time, by which life [is] governed’ (Wakefield and Braun, 2014: 5). It enacts governance through ‘anticipating, provoking, achieving, and consolidating’ (Legg, 2011: 131), while the urgent need determines how a dispositive forms. In the broadest sense, our understanding of dispositive refers to a particular set of relations that perform a defined function; both ‘the *set of elements* and [. . .] the *connections established between them*’, which have ‘no common dimension other than the urgency to which they respond’ (Braun, 2014: 51–52). As such, the dispositive is a relational network composed of ‘discursive and nondiscursive elements, material and semiotic entities, without any neat separation between them’ (Lemke, 2021: 92).

A common rationale runs through what we understand metabolic dispositives to be: one that emerged contemporaneously with the shift from industrial to post-industrial agriculture (Blanchette, 2020; Folkers and Opitz, 2022; Landecker, 2013b), whereby an exclusive focus on productivity became infused with a broader set of concerns about the regulation of risks and environmental overflows. The dispositive, here, entails a cybernetic-style engagement with systems-thinking, connectivity, and feedback loops (Clarke, 2020; Cooper, 2008); a framing that allows metabolic processes to emerge as a means of tweaking broader material flows. This amounts to an oscillating form of biopower that is applied simultaneously to bodies, populations, and the environments they inhabit. While metabolic processes have long been subject to governmental control (e.g. the diagrammatic food pyramids designed to inform consumers about diet and health; see Ibáñez Martín, 2018), bodies and populations have tended to be both the means and ends of a given intervention. Bodies and their metabolisms, in such cases, are understood as the route to better health. In the metabolic dispositives we’re interested in, by contrast, metabolising bodies are seen as the proximate means through which other material-discursive goals can be achieved.

In the nitrogen case, the proliferating usage of synthetic fertilisers and imported compound animal feeds has allowed for more intensified food production (Haalboom, 2022), but nitrogen compounds also pollute surrounding soils and waters, decreasing biodiversity and water quality. EU habitat directives thus place limits on nitrogen deposition in Natura-2000 areas, while water quality directives define standards for acceptable nutrient composition in surface water and groundwater. In the Netherlands, where intensive livestock farming predominates and governments and farming communities have postponed and resisted stricter regulations, environmental action groups have recently (and successfully) aimed to force stronger policies through landmark court cases, causing legal and social struggles that have become known as the ‘Dutch nitrogen crisis’. This crisis, in which cows are cast as central matters of environmental concern (Vogel, 2025), intensified a national and regional administration of animal waste and spurred the development of farm management techniques to make nutrient use on farms more efficient.

In the case of the carbon cycle, bovine metabolisms have become problematic owing to the greenhouse gases produced by the multispecies interactions that constitute bovine digestion. This problematisation is itself shaped by the way climate change has been conceived of by powerful actors as a technopolitical phenomenon in which carbon emissions can be accounted for and ‘solved’. Where in the case of the nitrogen cycle, cows emerge as metabolic pressure points in a specific space, in the carbon case, bovine metabolisms are cast as having excessive impacts that are felt on the planetary scale. As climate change is framed as a global problem requiring similarly global solutions, the governance of carbon emerges as a scaling challenge. The effort to align institutions, norms, and regulations towards political action before it is ‘too late’ is visible in the form of international pledges such as the Global Methane Pledge.^1^ Where the nitrogen problem so far hardly engages consumers’ dietary choices, the carbon problem bestows environmental responsibility onto the consumer via the discursive construction of so-called ‘low-carbon’ meat and dairy products (Folkers and Opitz, 2022).

In both cases, the scrutiny directed towards bovine agriculture’s environmental impact subjects cattle to emergent regimes of biopower aimed at optimising their metabolisms (McGregor et al., 2021; Folkers and Opitz, 2022; Ormond, 2020). Against this backdrop, and in a similar way to how Deleuze (1992) describes the dividuation of subjects into ‘dividuals’, metabolo-politics involves the dividuation of animal bodies into a set of interlinked metabolic processes. Individual animal bodies are thus disaggregated into metabolic processes, like methano-genesis, before being re-aggregated and reimagined as composite bodies, like herds (Buller, 2013). In both the nitrogen and carbon cases we examine, it is the instantiation of a bovine herd (at the level of the farm, region, country, or planet) and ‘its’ metabolism that is problematised. In our cases, cattle are identified as the most targetable phase in a series of ‘interlocking cycles’ of metabolism (Landecker, 2013a: 193), situated across locations, water systems, atmospheres, bodies, populations, and other spaces. Metabolo-political interventions into individual bovine bodies thus figure as a proximate means through which other flows, other bodies, and other populations can be accessed and governed.

Scholars have demonstrated how governing environments via such proximate modes challenges how responsibility for ecological impact is determined (Lamoreaux, 2016). Indeed, the metabolic dispositive we are describing is conditioning a new agenda for agricultural management and environmental ethics (Cusworth, 2023; Hinchliffe et al., 2025), and changing how bodies and populations, insides and outsides, are understood by agricultural practitioners and environmental policymakers. On farms, where knowledge of bovine digestive processes was historically leveraged to increase production, cows are now also being engaged as levers to tweak the flow of agricultural pollution into landscapes or to manage the risk of agricultural disease (Cusworth and Lorimer, 2024). Metabolic processes are now ‘suffused with environmental risk, regulation, and information’ (Landecker, 2013b: 497). Metabolo-political interventions thus respond to different environmental crises and their associated regulations (e.g. biodiversity loss or climate change) that have been caused, in part, by the cumulative effects of previous rounds of metabolic intensification (i.e. industrial agriculture).

These new logics entail a partial shift from *the government of things* to *the government of circulations*. Metabolo-politics is thus not a break with biopolitics but an active recognition of how *biopower operates in oscillatory motion* between individuals (anatomo-politics) and populations (biopolitics), and how that motion allows certain actors to access target flows and problematised ambient conditions.

## Metabolic Milieus

In metabolo-political interventions, the metabolic processes of individual bodies figure as a proximate means of accessing and modulating population-scale metabolisms that are themselves a proximate means of intervening in the material conditions those bodies both inhabit and help form. Biopower, to be sure. But biopower both *in* and *through* the ambient conditions and networked infrastructures of life (Barua, 2025a), which constitute the *metabolic milieu.* The milieu has been described as ‘the environment, the context, of an organism’, that allows it ‘to live more or less effectively’ (Elden, 2019: 21). Scholars have applied Foucault’s notion of the milieu – in conversation with the work of his mentor, Georges Canguilhem (Canguilhem and Savage, 2001) – to diverse contexts including viruses (Greenhough, 2023) and chickens (Barua, 2025a). This work has entailed a shift away from conceptualising human-nonhuman relations as dyadic interactions between two discrete entities (e.g. human-cow), towards accounting for a broader set of polyadic relations in a given emergent territory (Deleuze and Guattari, 1987). In this section we conceptualise the metabolic milieu as the spatialisation and scaling of a given metabolic dispositive with attention to the political implications of its representations and associated discourses.

The metabolic dispositive plays an important part in determining which aspects of the infinite and churning material totality come to form a specific milieu: which ‘transversal’ (Barua, 2025a) movements and which metabolic transformations are relevant to an *urgent problem*, and thus which nodes are salient for delivering its governance, in a given space and scale. Indeed, the metabolo-political logics differ in our two cases, enacting different spatial (global and local) and temporal (short- and long-term) scales, and involving different actors. In the carbon case, the metabolic milieu is constituted by the carbon that moves through the livestock sector, along with all the lifeforms, materials, discourses, and forces it encounters as it circulates. And in the nitrogen case, the metabolic milieu is constituted by materialities including the synthetic nitrogen fertiliser which is imported from Eastern Europe, the compound feedstuffs imported from South America, the bovine rumen the nitrogen passes through, the muscle the nitrogen helps grow, the excretions of metabolising bovine bodies, and the husbandry infrastructures that determine how manure decomposes into compounds that bioaccumulate in local nature reserves. The carbon case is imbued with the epochal temporality of climate change, whereas the nitrogen case’s temporality is more closely aligned with Dutch political cycles and the traffic of nutrient pollution from agricultural landscapes to local waterways. In both cases, the metabolic milieu extends beyond the immediate spatiotemporal surroundings of bodies, encompassing a dispersed set of material relations (Cusworth, 2023).

Focus on the milieu draws attention to how metabolo-politics is always contextual and situated, where environmental biopower operates through area-specific governmental and vernacular logics. In both cases, though, attention is directed not just to the agricultural outputs of bovine metabolism (meat and dairy), but also their byproducts (water pollution, methane, local biodiversity impacts). This is biopower in the aftermath (Landecker, 2019), in which the post-industrial agricultural sector is concerned not solely with managing growth and productivity but mitigating against that productivity’s ‘critical mass of side effects’ (Beck et al., 2003: 2). The problem with these side effects is the difficulty encountered when trying to manage them. Flows and cycles are diffuse. They cannot be grasped directly in the way that a body might be subject to restraint or containment. Metabolo-political strategies target the metabolisms of individual bodies and aggregate populations as a means of targeting the wider environmental surroundings those metabolisms help form (Folkers and Opitz, 2022).

Disciplinary strategies emerge in relation to the spatiality and scale of a given metabolic milieu. Sometimes, disciplinary strategies compel the inclusion and exclusion of bodies from particular spaces. In the case of ‘low-carbon’ cows, the farmers servicing contracts for Burger King net-zero burgers are compelled to ensure that their farm contains the breeds and feedstuffs needed to create the metabolic configurations capable of delivering on low-carbon marketing promises. Other interventions into the metabolic milieu, however, resemble what Deleuze (1992) refers to as control: light touch exertions of power that modulate rather than mould, encourage rather than compel. Here, governance ‘is as much about managing circulation and modulating flows as it is about molding individuals’ (Wakefield and Braun, 2014: 5). In the case of nitrogen pollution in the Netherlands, even in the face of a pressing legal need to drastically reduce nitrogen emissions to bring them in line with EU legislative limits, the Dutch national and provincial governments and industry actors have avoided coercive methods (such as closing farms), instead introducing voluntary buyout schemes to encourage shifts in farm management. These strategies avoid directly disciplining target bodies and instead involve the curation of economic incentives that are designed to reformat metabolic worlds in desirable ways.

The diffuse nature of metabolic milieus means that animal bodies are often chosen as the site of metabolo-political intervention. Through corporeal intervention, metabolo-political power operates by regulating the milieu, both by enforcing borders and by ‘making possible, guaranteeing, and ensuring circulations’ (Foucault, 2007: 29). Whilst control cannot be exerted directly over the carbon or nitrogen cycles themselves, bovine bodies *can* be subjected to forms of biopower that render those material circulations strategically tractable. This is because bovine bodies represent a momentary stabilisation of complex material flows. They become passage points for nitrogen and carbon, and discrete nodes of activity in which these elements are transformed and excreted.

The diverse strategies available to the operators of metabolo-political power do not necessarily lead to total and exacting precision. Owing to the complexity of the milieu being intervened in – as well as the variation and unruliness of the metabolic processes being used to access it – metabolo-political interventions deal in approximations and averages. They govern towards ‘variation[s] within a range’ (Barua, 2025a: 4) rather than fixed norms. In the Netherlands, expert committees have formulated ‘critical loads’ (*Kritische Depositiewaarden*) for each EU-defined habitat type – the thresholds after which nitrogen deposition negatively affects forms of plant and animal life (e.g. in Dutch nature reserves). These critical loads are then used to define the legal ‘*stikstofruimte*’ – or ‘nitrogen space’ – for further economic activities in the target area. This nitrogen space is calculated by subtracting the current deposition rates from the determined critical load thresholds. These calculations are regularly refined based on updated ecological field studies and models and they render certain aspects of nitrogen’s material circulations legible to governance, turning metabolic processes into a calculable budget. The changeability of these nitrogen budgeting calculations demonstrates how measurements and targets are always contested, dynamic, and articulated differently by different actors (Foucault, 1977; Cusworth et al., 2022a).

The material properties of a milieu are therefore never separate from the discourses and representations that shape the spaces and styles of metabolic intervention. For this reason, many forms of metabolo-politics are aimed at the human nodes of the metabolic milieu. Food marketers, for example, prime climate-anxious consumers with information about the (purported) emissions savings made available through metabolically-altered husbandry systems to coax them into making specific eating decisions. In the Dutch nitrogen case, different politicised accounts of the origins of nitrogen pollution and its rightful solutions circulate, and they recruit different portions of civil society. The 2019 farmer protests, which were sparked by the nitrogen pollution crisis introduced above, for example, sought to make visible the animal welfare compromises and the undue economic and emotional stress the farming community would suffer as a result of the government’s proposed pollution regulations. These demonstrations also sought to emphasise the crucial role farmers play in caring for cows and the land, and in achieving food security goals – the latter manifest in the protest slogan ‘no farmers, no food’.

Even in the two relatively similar cases explored here, where both feature technoscientific and market-friendly interventions into bovine bodies and their environments, the highly area-specific, situated character of metabolo-politics is on full show. In the more materially dispersed and inter-scalar case of carbon, international climate governance mechanisms, paired with a broadening public concern about the environmental impacts of human diets, are inaugurating interventions targeting the methano-genesis process that unfolds in the bovine microbiome. In the case of nitrogen, local biodiversity concerns are interacting with EU-, national-, and regional-level political responses, and they are inaugurating new forms of on-farm business management, infrastructural development, and civil society unrest.

Owing to the dispersed materiality of metabolic milieus, metabolo-political actors must call on diverse methods and technologies when intervening in a milieu. In the next section, we turn to the various types of metabolic technologies that emerge through distinct knowledge practices, discourses, and economies.

## Metabolic Technologies

Throughout his work, Foucault uses the term ‘technology’ to capture the myriad discursive, ideological, material, and scientific means through which power is articulated (Behrent, 2013). Whilst technology *can* refer to the artefacts and machines that the word invokes colloquially, the term is not limited to such things. Signage systems, semiotic codes, governance frameworks, rules, regulations, societal expectations, and other modes of (self-)discipline are all constituent parts of the technology and techniques of government (Foucault, 1997). They enact the milieu as something materially, semiotically, and discursively tractable; exposing it to different political, economic, and cultural interventions. The use of metabolic technologies also actively and iteratively reformulates urgent problems, making them legible in new ways and to new publics. In this section, we describe the forms these technologies can take under four operational themes as they shape metabolo-political practices: mapping, manipulating, multiplying, and messaging.

*Mapping* refers to the process of making the metabolic milieu legible diagrammatically. Our use of ‘diagram’ here is not limited to visual abstractions but rather, drawing on Deleuze’s (1988: 35) reflections on Foucault, an ‘intersocial and constantly evolving . . . model of truth’. Diagrams work through ‘unmaking preceding realities and significations, constituting hundreds of points of emergence or creativity, unexpected conjunctions or improbable continuums’ (Deleuze, 1988: 35). Approaches to mapping bovine metabolisms have origins in industrial ecology and its diagrammatic focus on the inputs and outputs of metabolic systems (Fischer-Kowalski, 1998; Gandy, 2025; Newell and Cousins, 2015). This mapping involves detailing the layered multispecies intra-actions that define how specific materials – here, carbon and nitrogen – move in, through, and out of bovine bodies and into wider environments. Metabolic technologies, therefore, do not just make biochemical interventions into pre-defined milieus but actively perform milieus as actionable in space and time.

In the case of methane-reducing cow feed, microbial interactions in the rumen are studied to understand how bovine bodies transform feedstuffs into muscle mass and methane. Agri-tech companies conduct metagenomic analyses of host-microbiome relations in an attempt to determine which microbial metabolisms produce methane (Clemmons et al., 2019). Specific prokaryotes (e.g. *Methanobrevibacter ruminantium*) are then indexed to lower-than-average rates of methane production, whilst others (e.g. *Methanobrevibacter gottschalkii*) are linked to higher rates (King et al., 2011). In the Netherlands, dairy companies now benchmark the urea content of milk – an indicator of the efficiency of a cow’s raw protein uptake and ammonia excretion – so farms can be compared between each other and over seasons, with the aim of eventually improving protein efficiency and reducing nitrogen runoff as ammonia or nitrate.

Metabolic maps inform metabolic *manipulations* dependent on complex multispecies interactions: nitrogen is made available to crops through diverse prokaryotic metabolisms, whereas methanogenesis and other metabolic pathways are enacted by ruminal archaea in anoxic conditions, symbiotically inhabiting bovine bodies. Manipulations are interventions into metabolic processes designed to shape how target metabolisms interact with the wider flows and environmental circulations they help form. The goal here is ‘to intervene in a system of interlinked processes by making few but key adjustments’ (Vogel, 2025: 75). Agri-tech companies design feed additives that interact with the bovine microbiome to produce less methane as the bovine holobiont metabolises its feed; just as farmers, government officials, and agricultural research groups are developing cattle sheds, nutrient management infrastructures, and riparian buffers to reduce nitrogen runoff (Vogel, 2025). Importantly, the methods, scales, and logics of these mapping and manipulating technologies depend on the materiality under scrutiny. For instance, water pollution issues demand methods that can attend to, and intervene in, the relatively viscous and macroscopic flows that determine how microscopic nutrients travel out of farms and into local water bodies.

There is major power invested in the ability to design and conduct the use of these technologies. In the case of methane-inhibiting feed additives, there are dissenting voices within the research community that suggest that methane production rates return to baseline levels after sustained periods of feeding with ‘climate-friendly’ feed additives (Searle et al., 2024). The point is that the ability to decide how to ‘map, measure and diagram the pathways of nutrients’ (Barua, 2025a: 10) – in this case by selecting an experimental time horizon that captures the desired impact of the additive but ignores its long-term inefficiency – amounts to a (covert) ability to decide which interventions emerge as desirable (Cusworth et al., 2022a). Powerful actors involved in performing a more sustainable livestock industry are, in this way, able to make ‘some units of animal production visible as central [to sustainability] while ignoring or downplaying others’ (Hastrup et al., 2022: 5584). These performative representations serve not only to substantiate and legitimate specific actions but to occlude the value of pursuing alternative governmental strategies (Cusworth and Stanley, 2025) like the de-animalisation of the food system.

Metabolic maps contain important information about *multiplication*; about how, and with what effect, local or individual metabolic changes can be scaled up or scaled down. Industry actors, for example, use cow-level assessments of their climate-friendly feed additives to calculate the methane reductions that might follow from using those additives in whole herds of animals. So too are farm-level changes in nitrogen management practice indexed to local or national-scale environmental improvements. These economistic logics – derived from input/output accountancy-style calculations – characterise the metabolo-political interventions studied here. They chart a path that the oscillating logic of metabolo-politics can follow: from the metabolic processes of individual bodies or individual farms, to the metabolism of a collective, and through that collective, to the wider environmental milieu that is indirectly accessed. Such mapping is a vital part of the metabolo-political infrastructure needed to visualise and account for – and thus render governable – target metabolic processes (Legg, 2005, 2006).

The usage of methane-inhibiting feedstuffs does little to change the macro-scale flow of carbon in, through, and out of the livestock sector until those products are widely implemented. Equally, the adoption of nutrient management practices on single farms does little to tackle national crises. As a result, to multiply the impacts of metabolic manipulations, the activities and behaviours of a sufficiently large cohort must be coordinated. This topology marks a distinct feature of the metabolo-political technologies we are describing, whereby interventions are leveraged to change not only populations (of humans or nonhuman animals), but rather the biochemical flows that constitute the broader conditions of life. In our cases, the metabolic processes of individual bodies, or even the cells they are composed of, are one extremity in the oscillating exercise of biopower via bodies, populations, and ambient environmental conditions. They are the proximate levers through which the global flow of carbon or the regional flow of nitrogen can be administered. Metabolo-politics as a distinct mode of biopower thus hinges on the ability to multiply metabolic manipulations outwards.

Metabolic *messaging* plays an influential part in this multiplication process. Proposed interventions are distributed to farmers in the form of low-carbon cow breeds, feed additives, and technological infrastructures designed to capture the gases and nutrient overflows produced by bovine rumination. In the case of low-carbon burgers, feed additives designed to reduce bovine methanogenesis are set to become part of environmentally-conscious consumer demands. These strategies can be read as an attempt by industry actors to defer regulatory intervention (e.g. by state actors) by demonstrating that the sector can responsibly manage its own environmental impacts autonomously (Morris and Jacquet, 2024). This messaging is intended to encourage the establishment of certain metabolic interventions in a broader set of bovine bodies throughout more and more farms, to multiply and scale the material impact of intervention – thus increasing public demand for their product.

Metabolo-political *messaging* can be understood as an expression of Foucault’s (1988) ‘technologies of the self’. From the perspective of industry actors, the hope is that by representing and advertising the manipulations being made into bovine rumina, consumers and other downstream food actors will ‘self-regulate’ by choosing their low-carbon meat and dairy products. Consumers are framed as having the power to effect a food sustainability transition through their consumption choices (Sexton, 2018). Agrawal’s (2005) notion of ‘environmentality’ thus remains key to certain instances of metabolo-politics: it is through the subjectifying power of the metabolic dispositive that metabolic subjects come into being, and it is (at least partly) through their self-disciplining technology that metabolo-political ends are framed as achievable. Obvious links to greenwashing are apparent here (Cusworth et al., 2022b) as these interventions frequently involve securing the status quo by governing public opinion rather than achieving structural changes in material conditions. In other words, metabolo-political subjectification occurs regardless of material impact. Indeed, we do not suggest that consumer agency is primary in metabolic governance, given the myriad and often nefarious discursive interventions which shape ‘consumer choice’, from advertising to nudge economics.

Gabrys (2014: 32) presents environmentality ‘not as the production of environmental subjects but as a spatial–material distribution and relationality of power through environments, technologies, and ways of life’, where individuals are recast as nodes in a system. In this sense, ‘government today seeks to modulate flows *and affects* as much, or even more than, it seeks to produce subjects who understand and relate to themselves in a particular manner’ (Wakefield and Braun, 2014: 7, emphasis added). In the context of metabolo-political power, the relative weighting of the logics of self-discipline, affect, subjectification, and control is highly context dependent. Where power is more consolidated and where the behaviours of a smaller number of actors need to be coordinated, the self-disciplining subjectifying component will be of less relevance (closer to the Dutch case, where there was broad sectoral interest in nitrogen regulation but relatively modest public engagement). Where outcomes emerge as the result as a function of aggregated behaviours, the allure of metabolo-political subjectification stands to be much greater (as in the low-carbon burgers and attempts to cultivate environmentally-conscious consumers).

These four operations – mapping, manipulating, multiplying, messaging – iteratively condition one another in layered and non-sequential ways. New research into the development of climate efficient bovines and pollution-suppressing on-farm management practices is used to create maps of emissions and emission reduction opportunities on the farm. Those maps can be used to evaluate and enact meaningful ecological improvements. They may also generate more hype, interest, and speculative investment for future experimentation and action, or placate those who might otherwise be concerned about the polluting outcomes of animal husbandry systems (Brice et al., 2022; Ormond, 2020; Sexton et al., 2019). In combination, metabolic technologies create what we describe as the oscillating action of metabolo-politics: the movement of power back, forth, through, and between different bodies, actors, and material flows.

## Metabolo-politics in the Aftermath

This article conceptualises metabolo-politics as a contemporary mode of environmental biopower operating through material flows, transformations, and circulations. Our analysis has coalesced around the ways bovine bodies are figured as nodes in the governance of broader biogeochemical cycles. Metabolo-politics operates as an oscillatory mode of biopower, between bio- and anatomo-politics. In such contexts, bodies help access populations, populations help access diffuse flows, and diffuse flows can be changed to alter the environments those (and other) bodies inhabit. Lemke offers us three concepts for apprehending how biopower works environmentally: dispositive, milieu, and technology. In conceptualising metabolo-politics, we develop Lemke’s Foucauldian framework in an environmental and agricultural mode. Our contribution to the contemporary wave of metabolic thought highlights the relationship between these three concepts. To re-emphasise, metabolic dispositives, milieus, and technologies iterate one another, without one preceding the other.

To identify metabolo-politics as a motif of contemporary environmental governance, we draw from case studies in post-industrial agriculture in the Global North, primarily in Western Europe. We do this through a focus on how bovines and their ecologies become central nodes in the governance of two biogeochemical cycles heavily impacted by industrial activity: carbon and nitrogen – both cycles that have wide-reaching implications for the liveability of multispecies worlds. By attending to the different metabolo-political logics at work in two similar cultural, economic, and ecological contexts in which bovine metabolisms are the focus of environmental governance, we have illustrated how metabolo-politics is *always situated*, i.e. its logics are always context-dependent and variegated, rather than totalising and universal.

Our metabolo-political framework, which was generated through research on the UK, US, Switzerland, and the Netherlands, will benefit from examination and alteration across other ecological, historical, and cultural contexts pertaining to the metabolic governance of life. As ecomodern technofix ideologies are practised by agricultural actors in diverse geographies, they are met with different ontological and epistemological assumptions regarding the governed nonhumans and environments in question, and the logics by which they should be administered. Again, environmental biopower is always situated, and metabolo-political logics produced in powerful technoscientific hegemonies are diffracted as they travel across global networks and are implemented elsewhere. Attention to these variations is essential if our metabolo-political framework is to have analytical purchase beyond these empirical cases. While this article has revealed significant differences in metabolo-politics from two situated contexts in Western Europe, we call for the further provincialisation of this concept in future research (see Davies, 2019; Meloni, 2020).

Across contexts, however, a shared concern with the impacts globalised industrial agriculture is having on the worldwide web of life invites examination into the specifics of how new forms of governance emerge in ‘the aftermath’ of anthropogenically-induced biological change (Landecker, 2025). There remains a potential theoretical tension here. Landecker (2025: 680) explicitly contends that whilst living through the blowback of anthropogenic interventions in biology ‘one might well be tempted to declare not biopower today, but biofallibility today’. And so, while the techniques of industrialised agriculture may have led to numerous unpredicted environmental overflows, such overflows are nonetheless instructive. They reveal a history of *omissions* alongside emissions. But they also point to a future of living and governing in the aftermath. Contemporary environmental governance, then, is always just as much about responding to a set of conditions as it is about creating new ones. Seen in this historical context of omissions, the metabolo-political interventions we describe and analyse in this article can be read as an attempt to make sense of the material excesses of agricultural intensification and capitalist acceleration in Western European livestock agriculture.

The point here is that the location and character of these barnacles on the hull of modernity might reasonably prompt us to speculate on where the next ruptures might emerge. We contend that despite the diagnosis of biofallibility in the aftermath of intervention – as raised by Landecker (2025) – the actors discussed throughout this article (and more generally engaged in the metabolic government of life) justify their metabolo-political interventions in the belief that these interventions will work. It is worth quoting Landecker (2025: 701) at length here:

Comprehension of biological power, fallibility, and their interrelation calls for different conceptual work by which we can recognize that techniques of knowledge and control that unwind the facts of life can work perfectly well, be powerful, and know and do many of the things they were meant to, at least for a while or at least open out new futures for experimentation, and can at the very same time have a supplemental set of growths, a second crop, that comes about at different speeds and scales, becoming legible sometimes sooner, sometimes later.

We offer metabolo-politics as one such form of conceptual work to reckon with this complex interrelation of biopower and biofallibility. We wonder, then, what is being overlooked or omitted in the emerging metabolo-political efforts to manage the food system’s environmental impacts. In the case of the climate-friendly cows and the development of methane-suppressing feed additives, such omissions (and their outcomes) are beginning to come into focus. Concerns are being raised about the animal welfare risk of bloating and the discomfort experienced by bovine bodies whose metabolic processes are being tinkered with in the name of climate mitigation (Kjeldsen et al., 2023). Thus, whilst the production of methane in the bovine rumen might be seen by food system actors as either a byproduct to be contained or an indicator of an inefficient nutrient conversion rate to be optimised, methano-genesis is also an important part of the holistic way bovine bodies eat, digest, and excrete in ways that are comfortable and efficient *for them*.

We raise these concerns not (just) to highlight the animal welfare consequences of using bovine rumina as a site of planetary geoengineering. We raise it to stress that even distended frames of action and knowledge – such as those employed in metabolo-political governance – have their partialities (Cusworth, 2025). And, further, that these oversights and omissions will play an important role in determining how the harms and benefits of contemporary governance intervention will be distributed. In the logics of climate governance, animal welfare outcomes risk being incommensurate (Brice et al., 2022): an incommensurability that is instructive of the way things get left out as certain problems are allowed to become urgently problematic and as certain nodes are allowed to become actionable. There is power, then, invested in the very ability to depict something as being the sort of flow or cycle that is amenable to a particular type of (metabolo-political) intervention (Hopwood et al., 2021). For this reason, metabolo-political interventions distribute vulnerabilities unequally. Studying environmental biopower in the aftermath thus requires attention to the mechanisms by which certain metabolic relations become hegemonic while alternatives are foreclosed. It requires attention to those metabolisms that are framed as nourishing, and those that are framed as destructive or inefficient – and for whom.

Across these various omissions and aftermaths, our hope is that the heterogeneous elements we have invested into the concept of metabolo-politics offer a workable and adaptable framework for pursuing the logics and consequences associated with the metabolic governance of life and matter in circulation as it unfolds across difference.

## Notes

1. See: https://www.globalmethanepledge.org/ (last consulted 12 October 2024).
